# High Frequency of Glucose-6-Phosphate Dehydrogenase Deficiency in Patients Diagnosed with Celiac Disease

**DOI:** 10.3390/nu14091815

**Published:** 2022-04-26

**Authors:** Maria Pina Dore, Alessandra Errigo, Stefano Bibbò, Alessandra Manca, Giovanni Mario Pes

**Affiliations:** 1 Dipartimento di Medicina, Chirurgia e Farmacia, University of Sassari, Viale San Pietro 8, 07100 Sassari, Italy; s.bibbo@gmail.com (S.B.); al_manc@yahoo.it (A.M.); gmpes@uniss.it (G.M.P.); 2 Baylor College of Medicine, One Baylor Plaza Blvd, Houston, TX 77030, USA; 3 Dipartimento di Scienze Biomediche, University of Sassari, Viale San Pietro 43B, 07100 Sassari, Italy; a.errigo@studenti.uniss.it; 4 CEMAD Digestive Disease Center, Fondazione Policlinico Universitario Agostino Gemelli IRCCS, 00168 Roma, Italy; 5 Sardinia Longevity Blue Zone Observatory, 08040 Nuoro, Italy

**Keywords:** celiac disease, antioxidant defense, glucose-6-phosphate dehydrogenase deficiency

## Abstract

Celiac disease (CD) is characterized by a proinflammatory state associated with the production of reactive oxygen species, i.e., a condition of oxidative stress. In this study, we tested the hypothesis that the inherited deficiency of glucose-6-phosphate dehydrogenase (G6PD), by causing impaired antioxidant defense, may increase the risk of CD. Methods: A retrospective monocentric case-control study was performed using the clinical records of 8338 outpatients (64.6% women) scheduled for upper endoscopy between 2002 and 2021 in Northern Sardinia. Overall, 627 were found to have CD (7.5%), and 1027 resulted to be G6PD-deficiency carriers (12.3%). Since randomization was impractical, the potential covariates imbalance between cases and controls was minimized using a 1:2 propensity-score-matched (PSM) analysis. Results: Overall, G6PD deficiency was associated with increased risk of CD (odds ratio (OR) 1.50; 95% confidence interval (CI) 1.19–1.90). The PSM procedure identified 1027 G6PD-deficient and 2054 normal patients. Logistic regression including the propensity score detected for G6PD deficiency an OR of 1.48 (95%CI 1.13–1.95; *p* = 0.004). Conclusions: Our findings show that the enzyme defect was significantly and positively associated with CD, in line with the pro-oxidant impact of the enzyme defect observed in animal models and humans.

## 1. Introduction

Celiac disease (CD) is a chronic, immune-mediated enteropathy triggered by the ingestion of gluten-containing foods in genetically predisposed individuals [1]. In recent decades the incidence rate of the disease has risen both in advanced and developing countries, and it is currently estimated to affects around 1% of the global population, implying consistent medical care expenditures [2]. Similar to other inflammatory diseases, CD shows a marked gender disparity, being more frequent among females, especially in childhood [3,4]. The pathological hallmark of CD is a diffuse lymphocytic infiltration of the small intestinal mucosa, leading to enterocyte damage and apoptosis. The pathogenesis of CD is only partially known. Gluten peptides contain proline-rich epitopes resistant to the action of gastrointestinal proteases [5]. Peptides containing gliadin filter through enterocytes due to increased permeability mediated by the direct binding of gliadin with CXCR3 chemokine receptor, located in the tight junctions [6]. The peptides, made immunogenic through deamidation operated by the tissue transglutaminase, bind to the HLA-DQ2/8 molecules in the antigen-presenting dendritic cells, triggering an adaptive Th1 immune response involving the CD4+ T cells [7]. These activated CD4+ T cells release various pro-inflammatory cytokines, including IFN–γ, interleukin (IL) 2, IL-4, IL-15, and TNF–α [8]. The presence of IL-17-produced by CD4+ T lymphocytes has also been described in untreated CD patients, but its precise role has yet to be clarified [9,10]. The activation of intraepithelial lymphocytes in the inflamed mucosa depends on stress signals coming from the enterocytes themselves, such as, for example, the protein encoded by the MHC class I chain-related A (MICA) [11]. The immune responses to gluten may be downregulated by some infectious agents, such as *Helicobacter pylori*, the major causative agent of gastritis, apparently reducing the risk of the disease although the precise mechanisms involved are uncertain [12,13].

A number of studies both in vitro and in vivo have ascertained that oxidative stress plays an important role in the pathogenesis of CD [14,15,16,17,18]. In 1999, Rivabene et al. reported a lower level of reduced glutathione (GSH) and increased lipoperoxides in intestinal Caco-2 cells treated with wheat gliadin [16], an evidence confirmed by studies on intestinal biopsies of CD children [17] both treated and untreated [19]. Although the thiol GSH is involved in the main intracellular antioxidant pathway in the gut [20], this tripeptide is rapidly exhausted during the free radical detoxification and needs to be regenerated from glutathione disulfide GSSG through various enzymatic reactions, including the one of glutathione reductase [21] requiring the availability of the cofactor nicotinamide adenine dinucleotide phosphate (NADPH) as a reducing equivalent. The two main biochemical pathways providing NADPH are the mitochondrial malic enzyme and the pentose phosphate pathway (PPP), whose rate-limiting enzyme is glucose-6-phosphate dehydrogenase (G6PD). Accordingly, G6PD knock-out animal models show a disproportionate generation of reactive oxygen species (ROS) and tissue manifestations of oxidative damage [22].

On these premises, we conjectured that hereditary deficiency of G6PD might contribute to the immune damage of the intestinal mucosa in responsive subjects exposed to gluten through an impairment of the antioxidant capacity. To verify this hypothesis, we investigated in a case-control study whether G6PD-deficient subjects from Sardinia—a population where the frequency of the genetic defect is nearly 10% [23]—is associated with increased susceptibility to CD.

## 2. Materials and Methods

This was a retrospective case-control study performed on clinical records of patients scheduled to undergo endoscopy procedures in a teaching hospital of Northern Sardinia from 1996 to 2021. All patients were interviewed by a trained gastroenterologist who collected an accurate medical history. Demographic information, including age and sex as well as smoking habits and *H. pylori* infection, were retrieved and entered in a digital database. In the case of multiple exams for the same patient, only the index procedure was kept in the analysis.

### 2.1. Diagnosis of Celiac Disease

The diagnosis of CD was made according to the international guidelines progressively developed [24,25,26,27], and the majority of CD patients was clinically followed up in our gastroenterology service. Briefly, patients with gastrointestinal and/or extraintestinal symptoms suggestive of CD and/or with manifestations of malabsorption underwent a serologic testing. Serology was assessed in patients on gluten-containing diet. Patients with a high probability of CD underwent an upper endoscopy to collect duodenal mucosa specimens regardless of the endoscopic features. Small intestine samples were evaluated by a dedicated pathologist (A.M.) and intestinal lesions graded according to the Marsh–Oberhuber classification [28].

### 2.2. Helicobacter pylori Status

*H. pylori* infection was diagnosed through the histological examination of gastric biopsy, as previously reported [29]. In the case of a chronic-active gastritis and absence of the bacteria, the infection was confirmed by the stool antigen test or ¹³C-Urea breath test, as recommended [30].

### 2.3. G6PD Deficiency Assessment

The G6PD activity in red blood cells was measured using a G6PD quantitative kit following the manufacturer’s instructions [31]. Briefly, G6PD activity was measured by determining the rate of NADPH production. The G6PD and 6-phosphogluconate dehydrogenase (6PGD, an enzyme also producing NADPH) activities were measured, and the ratio was calculated. A ratio below 0.10 indicates total deficiency. Moreover, in a subgroup of CD patients, G6PD was genotyped. Since previous studies have ascertained that in the Sardinian population, there is a limited number of mutations associated with enzyme deficiency [32], the most frequent mutation (G6PD Med, 563 C → T, S188F) was screened, and in the case of negative results, the Seattle G6PD mutation (844 G → C, D282H) and G6PD Union (1260 C → T, R454C) were further assessed. Genotyping of G6PD 563C → T variant was performed by a PCR/RFLP method. The G6PD 563T allele introduces a new MboII restriction site and was detected by PCR amplification in exon 6 of the G6PD gene using the oligonucleotide primers 5′-AGGAGGTTCTGGCCTCTACT-3′ and 5′-TGAGGCTCCTGAGTACCACC-3′, followed by the digestion with the MboII endonuclease.

### 2.4. Statistical Analysis

Both descriptive and inferential analyses were performed. Results are expressed as means and standard deviations (continuous variables) or as absolute numbers and frequencies (categorical variables). Comparative analysis between normal and G6PD deficient patients was performed by the χ² test for categorical variables. According to smoking habits, patients were stratified into never smokers and current or former smokers. In order to minimize the different distribution of covariates between G6PD-deficient group and normal group so that the effect of G6PD deficiency on CD risk could be unbiasedly interpreted as a causal effect, a propensity score matching (PSM) analysis was performed. This procedure is often used to generate a suitable control group with observational data when randomization is impractical [33]. The PSM was implemented using the *MatchIn* package installed in the open-source R 4.1.2. software (http://www.rproject.org/, accessed on 12 February 2022), based on age, sex, area of residence, smoke, and *H. pylori* status. In total, 1027 patients with G6PD deficiency were matched with 2054 patients with similar PS using G6PD as the grouping variable. A 1:2 greedy nearest neighbors matching method was used, within PS calipers of ± 0.2 SD. Subsequently, the matched dataset was extracted to check if covariate (age, sex, residence, smoke, and *H. pylori* infection) balance was achieved, and the distribution of potential confounds was equal in both cases and controls. Finally, logistic regression analysis was performed with CD (presence/absence) as the main dependent variable and G6PD status as the main predictor variable, with PS included as a covariate [33]. Parameter estimates and their standard error (SE) for each covariate were calculated, as well as the odds ratios (ORs) and their 95% confidence intervals (Cis). The *p*-values lower than 0.05 were considered statistically significant.

## 3. Results

A total of 8338 clinical records of patients (64.6% females) who underwent upper endoscopy were available for the analysis. According to guidelines, based on histology and laboratory findings, 627 patients were found to have CD (7.5%). The distribution of age and sex among CD and non-CD participants is reported in Table 1. As expected, females were more prevalent in the CD group. Age at recruitment was lower in celiac patients (40.9 ± 16.3 years vs. non-CD: 52.7 ± 10.1 years; *p* < 0.0001). The proportion of CD patients from rural areas was significantly lower than those from urban areas. (34.6% vs. 50.8%, *p* < 0.0001). Current or former smokers were less represented among CD than in non-CD patients (39.4 vs. 47.3, *p* = 0.0001). As expected, *H. pylori* infection was less frequent among CD group than among non-CD group (32.9% vs. 56.4%, *p* < 0.0001) [13]. Notably, the G6PD deficiency was significantly more frequent in the CD group (15.6% vs. 12.0%, *p* = 0.0087).

As can be seen from the previous table, an imbalance of covariates prior to matching was evident concerning, in particular, the distribution of sex, smoking habits, and *H. pylori* infection, according to the standardized mean differences and variance ratios provided by the *MatchIn* procedure.

To make inferences more robust and less affected by the covariate imbalance, a 1:2 PSM was performed, which identified 1027 G6PD-deficient subjects and 2054 matched subjects with normal enzyme activity. Since G6PD deficiency is a, X-linked trait, the PSM procedure was applied in males and females separately in order to obtain sex-specific estimates of parameters. Table 2 reports the results of the 1:2 PS PSM procedure.

The probability of CD was estimated with G6PD deficiency as the exposure variable and adjusting for the other covariates, including PS as a continuous variable. An OR of 1.48 (95%CI 1.13–1.95; *p* = 0.004) indicated G6PD deficiency as an independent predictor of CD risk. The association was stronger in males than in females, according to an X-linked trait (Table 3).

**Figure 1:**
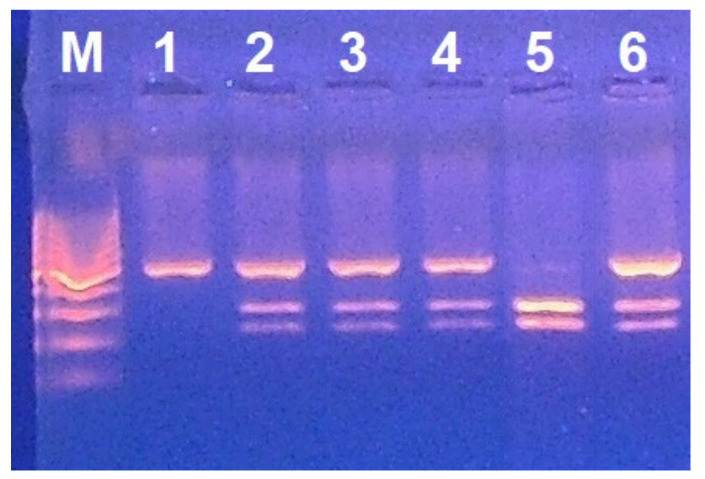
Identification of the G6PD Med mutation among CD patients by PCR and digestion with MboII enzyme. The mutated allele generates two fragments of 145 and 101 bp. M, marker; lane 1, normal; lanes 2, 3, 4, 6, heterozygote females; lane 5, hemizygote male (the PCR product band of 246 bp is absent).

Genomic DNA analysis was performed in 50 out of 627 CD patients and showed that five patients were carrier of the G6PD Med variant, located in the exon 6 of the gene (Figure 1). It was not found in the remaining CD patients without enzyme deficiency.

## 4. Discussion

The present study, conducted in a population with high prevalence of both CD and inherited G6PD deficiency, detected a significant association between the two conditions regardless of age, sex, smoking, residence area and *H. pylori* infection. Before applying the PSM analysis, our findings confirmed female sex to be a risk factor for CD, as previously reported [34]. Similar to observations present in the literature, cigarette smoking and *H. pylori* infection were inversely and significantly associated with CD [13,35,36]. When the strength of the association was tested in the two sexes separately, in males, the effect size was higher (OR 2.38), while in females, it was detected at a smaller amplitude (OR 1.22). This difference could be explained by the fact that G6PD deficiency is a typical X-linked trait; therefore, the affected males (hemizygotes) generally have total enzyme deficiency, whereas the affected females, excluding a few homozygote cases, are heterozygotes with only partial deficiency, likely still able to ensure adequate antioxidant protection. In a small subgroup of CD patients in whom the molecular analysis was possible, all mutations found in patients with G6PD deficiency resulted to be G6PD Med (S188F), a rather frequent variant in the Mediterranean area associated with severe enzyme deficiency (class II according to the WHO classification [37]). Consequently, at the moment, it is not possible to establish whether additional G6PD-deficient alleles, less frequent in the Sardinian population, are equally capable to increase the CD risk. Because CD prevalence decreases while oxidative stress increases with age, it could be argued that the association between the two disorders may be stronger in the elderly; on the contrary, in our cohort, the G6PD deficiency and CD were significantly associated before and after the age of 50. More specifically, the frequency of the enzyme defect remained fairly constant across all age groups, as previously reported [38], with a slight reduction in the last age decade.

Although the role of impaired antioxidant mechanisms in CD has been known for a long time [15,19,39,40], to our knowledge, this is the first study that correlates an alteration of PPP (a major antioxidant mechanism) and the risk of CD except for a case report [41]. In fact, in most populations, the frequencies of G6PD deficiency and CD are so low that it is rare to come across carriers with the double disorder. The rarity of this association makes it reasonable that the underlying molecular mechanisms have virtually never been investigated. In such circumstances, only a series of speculative hypotheses can be advanced while waiting for targeted molecular studies to bring additional evidence. In the Sardinian population, on the other hand, the frequency of CD is one of the highest in Italy [42,43], according to the fact that the frequency of HLA genotype predisposing to CD (DR3-DQ2) is 22.4%, second only to the Saharawi population (23%) across the world [44]. Moreover, the prevalence of G6PD deficiency is about 10% in the island [23,38], making the population of Sardinia ideal to test this association.

The G6PD is present in enterocytes [45], and it appears to be involved in the apoptotic process that is normally responsible for the continuous turnover of the intestinal epithelium. Some results obtained in cancer tissues originating from the digestive system suggest that G6PD can counteract the programmed cellular death, as demonstrated by the lower expression of apoptotic markers Bcl-2 and Bcl-xL [46]. Thus, it can be conjectured that the apoptotic mechanism boosted by cytotoxic T cells in CD is amplified in the enterocytes in which G6PD is dysfunctional. Early studies ascertained that the G6PD Med variant is a highly unstable protein, virtually absent in the mature red blood cells [47]. These findings may explain why enterocytes, with a short lifespan (4–5 days) comparable to that of reticulocytes (1–2 days), may undergo oxidative injury from the sudden depletion of G6PD [48].

Moreover, recently, there is growing evidence that G6PD deficiency impairs specific cytokine pathways involved in the immune response [49,50,51]. In experimental CD models, gluten exposure triggers the production of transforming growth factor-beta (TGF-β) [52], and increased expression of this cytokine has also been reported in the lamina propria of children with villous atrophy [53]. Given that G6PD-deficient mononuclear cells display increased expression of TGF-β [54], it can be hypothesized that this mechanism may contribute to intensifying the proinflammatory response to gluten, worsening the epithelial damage [55], although there is evidence in contrast with this hypothesis [56]. Discrepancies maybe due by different studies design. In fact, in transgenic mouse models it has been demonstrated that the TGF-β action is dual [57]. At systemic level its activity is mainly immunosuppressive, whereas at the local level, it tends to enhance inflammation [57].

Although the hypothesis tested in the present study was never evaluated previously, making our findings unique, nonetheless, several caveats need to be mentioned. First, it was a retrospective case-control study. Such studies do not allow to estimate causal relationship, and therefore, a propensity-score-matching analysis had to be used in order to partly bypass this drawback. An additional limitation may be the small size of G6PD-deficient subgroup confirmed by molecular testing even though discrepancy between genotyping and laboratory testing was not detected, making results reliable.

## 5. Conclusions

The CD occurs mainly in the pediatric age, worsening the life quality of children. Although in most cases, it is a relatively benign condition that can be treated by eliminating gluten-containing foods from the diet, a percentage of CD patients experience chronic symptoms that may be invalidating and, although more rarely, the occurrence of cancer with a considerable economic burden for the families and the health system [58]. Our findings underlay a novel molecular pathway potentially involved in the onset of CD that need to be confirmed in prospective studies in order to better define the role of G6PD deficiency in this disorder.

## Figures and Tables

**Table 1:** Descriptive statistics in 8338 study participants according to celiac disease.

Variable	No Celiac Disease (n = 7711)	Celiac Disease (n = 627)
Sex, n (%)		
Male	2826 (36.6)	125 (19.9)
Female	4885 (63.4)	502 (80.1) **
Age, n (%)		
<30	856 (11.1)	177 (28.2)
30–39	1096 (14.2)	126 (20.1)
40–49	1280 (16.6)	138 (22.0)
50–59	1456 (18.9)	87 (13.9)
60–69	1593 (20.7)	61 (9.7)
70–79	1122 (14.6)	33 (5.3)
≥80	308 (4.0)	5 (0.8)
Residence, n (%)		
Urban	3790 (49.2)	410 (65.4)
Rural	3921 (50.8)	217 (34.6) *
Smoke, n (%)		
Never	4066 (52.7)	380 (60.6)
Current or former	3645 (47.3)	247 (39.4) **
*H. pylori* infection, n (%)		
No	3361 (43.6)	421 (67.1)
Yes	4350 (56.4)	206 (32.9) **
G6PD ^1^ deficiency, n (%)		
No	6782 (88.0)	529 (84.4)
Yes	929 (12.0)	98 (15.6) **

^1^ Glucose-6-phosphate dehydrogenase; * *p* < 0.05, ** *p* < 0.01.

**Table 2:** Propensity score 2:1 between 1027 G6PD-deficient subjects and 2054 matched subjects with normal enzyme activity.

Variable	G6PD ^1^ Normal (n = 2054)	G6PD ^1^-Deficient (n = 1027)
Sex, n (%)		
Male	530 (25.8)	265 (25.8)
Female	1524 (64.2)	762 (64.2)
Age, n (%)		
<50	872 (42.5)	440 (42.9)
≥50	1182 (57.5)	587 (57.1)
Residence, n (%)		
Urban	1059 (51.5)	528 (51.4)
Rural	995 (48.5)	499 (48.6)
Smoke, n (%)		
Never	1092 (53.2)	560 (54.5)
Current or former	962 (46.8)	467 (45.5)
H. pylori infection, n (%)		
No	903 (44.0)	448 (43.6)
Yes	1151 (56.0)	579 (56.4)
Celiac disease, n (%)		
No	1917 (93.3)	929 (90.5)
Yes	137 (6.7)	98 (9.5) **

^1^ Glucose-6-phosphate dehydrogenase; ** *p* < 0.01.

**Table 3:** Propensity score matching 2:1 in males and females.

Variable	Males		Females	
	G6PD ^1^	G6PD ^1^	G6PD ^1^	G6PD ^1^
	normal	deficient	normal	-deficient
No. patients	530	265	1524	762
CD (%)	2.6	7.5	9.1	11.9
Parameters				
Estimate ^2^	0.049		0.023	
SE ^3^	0.015		0.013	
*t* value	3.256		1.770	
* p*	0.001		0.076	

^1^ Glucose-6-phosphate dehydrogenase; ^2^ Adjusted for age, sex, residence, smoke, and *H. pylori* infection; ^3^ standard error.

## Data Availability

Data are contained within the article; however, the data presented in this study will be available on request from the corresponding author.
